# Human papillomavirus (HPV) vaccine knowledge, attitudes, and uptake in college students: Implications from the Precaution Adoption Process Model

**DOI:** 10.1371/journal.pone.0182266

**Published:** 2017-08-07

**Authors:** Marie Barnard, Phillis George, Mandy L. Perryman, Lori A. Wolff

**Affiliations:** 1 Department of Pharmacy Administration, University of Mississippi, University, Mississippi, United States of America; 2 Department of Leadership & Counselor Education, University of Mississippi, University, Mississippi, United States of America; 3 Graduate School of Education, Fordham University, New York, New York, United States of America; University of Rhode Island, UNITED STATES

## Abstract

The purpose of this study was to examine human papillomavirus (HPV) and HPV vaccine knowledge, attitudes, and uptake in college students and to identify factors associated with vaccination status utilizing the Precaution Adoption Process Model (PAPM). The sample included 383 undergraduates from a public university who participated in February and March 2015. Students were emailed an anonymous online survey assessing knowledge, attitudes, and perceptions related to HPV and HPV vaccination, as well as their stage in the PAPM regarding vaccination completion. Significantly more females (47.3%) than males (15.8%) were vaccinated. While most students had basic knowledge of HPV, they had low perceptions of their susceptibility to contract HPV. Most unvaccinated students were in the early stages of decision-making related to vaccination. Campus health centers have an opportunity to increase HPV vaccination rates. This study indicates that students need prompts from providers, as well as education regarding susceptibility to HPV.

## Introduction

Human papillomavirus (HPV) is a highly prevalent sexually transmitted infection associated with increased cancer risks [1–2]. An estimated 79 million Americans are currently infected with HPV, with an estimated 14 million new infections developing each year, almost half of these in 15 to 24 years old [1,3]. Effective vaccines are available and are recommended for both males and females in the adolescent years, but uptake has been less than optimal [4,5]. Prior studies have identified barriers to HPV vaccination, but they have focused on parental concerns, as the initial recommendation is for vaccination in the adolescent years when parents make health care decisions for children [6–9].

Little research has been conducted to understand how college-aged individuals begin transitioning to making their own health decisions as they become independent, and this knowledge gap extends to how they make decisions related to HPV vaccination specifically. Barriers identified by parents include cost and time, as the vaccine requires multiple visits to a health care provider to complete the series [6,10]. Other barriers identified include concerns that vaccination will increase risky sexual behavior [7,11]. Despite the fact that most insurance providers, including public programs such as Medicaid and Children’s Health Insurance Program (CHIP), cover the cost of the vaccination and research has shown that HPV vaccination does not increase risky sexual activity, these barriers have remained years after the vaccination has become available.

The national guidelines call for catch-up vaccination for young adults who were not vaccinated during adolescence [5]. College campus health centers offer an opportunity to provide the recommended catch-up vaccination for those not vaccinated during adolescence. Vaccination barriers identified for adolescents, such as lack of health care coverage, convenient health center access, and ability to make independent health care decisions, are less relevant for college students [6–10;12–15]. Most colleges require students to have health care coverage and health clinics are readily available on most campuses, thus addressing two key barriers. From both a health care and a student affairs perspective, most college students are able to make their own care decisions privately, potentially removing barriers related to perceptions associated with sexual activity. Additionally, getting vaccinated for HPV is engagement in a health behavior. The college years are a critical period for the development of health behaviors and health decision-making processes [16]. An understanding of how long-term risks (i.e., cancer risks from HPV infection) impact engagement in preventive health behaviors such as vaccination in the collegiate years is needed.

Weinstein’s Precaution Adoption Process Model (S1 Fig) was developed to explain the process by which people adopt preventive behaviors to protect against a risk. The model has seven discrete stages from unaware to action and maintenance of a preventive behavior and has been recommended as valuable for understanding the stages an individual goes through in making a vaccination decision [17,18]. The model suggests different interventions that can be useful to move individuals from one stage to the next. Applying this model to college students’ HPV vaccination behavior may allow interventions to be tailored to the specific educational and/or motivational need to successfully move them forward, with a goal of moving beyond intention to action.

In an effort to address the need, this study sought to examine HPV vaccination awareness and uptake in male and female college students and to utilize the Precaution Adoption Process Model (PAPM) to assess how knowledgeable students are about HPV and HPV vaccines, evaluate concerns about vaccination, and identify factors associated with vaccination status.

## Methods

A cross-sectional online survey study design was utilized. We developed a survey to assess students’ knowledge, attitudes, and perceptions related to HPV and HPV vaccinations, as well as stage in the PAPM regarding vaccination completion. The survey included items developed in previous studies as well as additional items created for this study (S1 File). Knowledge was measured utilizing the 14 items related to HPV knowledge developed by Licth et al. for use with female college students [19]. Two items related to more recent advances in knowledge of HPV and cancer risks (i.e., oral and anal cancers) were added, as well as two items related to HPV and men. Perceived vulnerability and concerns relating to the HPV vaccine were assessed utilizing items from two prior studies, with several additional items created for this study [20–21]. Survey items to assess individuals’ stage in the PAPM were based on Weinstein’s items and adapted for HPV vaccination behavior for this study [17]. Vaccination behavior and demographic characteristics were also collected.

The survey was distributed via email to undergraduate students at a public university in the state of Mississippi and remained available for approximately one month. Analyses were conducted using SPSS (version 22.0). Descriptive statistics, chi-square tests, and independent *t*-tests (*p* < .05) were utilized to characterize the sample and to compare responses from female and male participants. A logistic regression model was developed to examine factors associated with vaccination status. Approval by the University of Mississippi Institutional Review Board was granted prior to the initiation of the study. Informed consent was provided by participants prior to survey completion.

## Results

### Participants

The 383 participants were nearly evenly distributed across the four undergraduate classifications (freshmen through seniors), with a mean age of 21.01 years. The sample was 70.2% female and self-reported ethnicity as 76.8% White, 13.1% Black, 8.9% Asian, and 2.2% other, a distribution similar to that of the institution as a whole. The majority of the students (71.3%) indicated that they had a primary health care provider (S2 File).

### HPV and HPV vaccine knowledge and awareness

Responses indicated that most students had heard of HPV (92.4% of females and 82.9% males; *p* < .015). Significantly more females were at least aware of the HPV vaccine compared to males (75.8% vs 56.2%; *p* < .001). The knowledge of HPV scale had a potential score range of 0 to 18. There were no significant differences on the knowledge scale between female and male respondents (females M = 11.87 ± 2.36, males M = 11.99 ± 3.01). Approximately 90% of respondents were aware that HPV is sexually transmitted, can be transmitted even when asymptomatic, and infects both men and women (Table 1). Male students reported the internet and school as the most common sources of information about HPV and the vaccine, whereas female students reported health care providers and school as their most common information sources, with school reported as the second most common. Significantly more female students reported being aware of a family member or friend who had been vaccinated compared to male students (43.4% of females and 26.7% of males; *p* < .001). Provider recommendation rates differed as well, with 62.4% of females compared to 21.6% of males reporting that they had been offered the HPV vaccine by a doctor or nurse (*p* < .001).

**Table 1:** HPV knowledge by gender.

HPV Knowledge Scale Items	% responded correctly	
	Females	Males
Genital warts are caused by HPV^a^	66.3%	69.8%
HPV can cause cervical cancer^a^	93.3%	88.7%
Abnormal pap tests may indicate that a woman has HPV^a^	88.8%	82.1%
HPV can cause penile cancer^a^	51.4%	52.8%
HPV is transmitted by skin-to-skin contact^a^	42.6%	53.8%
HPV infects both men and women equally^a^	88.9%	94.3%
HPV is sexually transmitted^a^	94.4%	91.5%
I can transmit HPV even if I don’t have symptoms^a^	90.9%	92.5%
Most persons with HPV have no visible signs or symptoms^a^	90.9%	84.9%
HPV can lay dormant in the body for years without symptoms^a^	92.1%	91.5%
There is a vaccine available to prevent HPV infection^a^	82.1%	74.5%
Condoms are not effective in preventing HPV^a^	35.9%	31.1%
There is no cure for HPV^a^	66.1%	70.8%
Most adults are infected with HPV^a^	30.7%	34.9%
HPV infection among men is rare	68.9%	69.8%
HPV can cause head and neck cancer	21.7%	35.8%
HPV can cause anal cancer	54.0%	59.4%
There is an HPV tests for men	28.0%	20.8%

^a^Original item in the Licht et al. knowledge scale [10]

#### Perceived susceptibility and concerns about the HPV vaccine

While the majority of students expressed concerns about the impact of HPV, they had relatively low perceptions of susceptibility to contract HPV, with less than a quarter of respondents agreeing that they are at risk for HPV or likely to contract HPV in their lifetimes. Concerns about the vaccine were explored, and the strongest concerns identified were related to family and friends finding out if the student were to get vaccinated, highlighted in Table 2.

**Table 2:** Perceived susceptibility and concerns about HPV and the HPV vaccine.

Perceived Susceptibility and Concerns	% agree or strongly agree	
	Females	Males
I am at risk for getting HPV^a^	21.9%	24.8%
I am likely to contract the HPV virus in my lifetime^b^	21.9%	18.1%
HPV would be a severe threat to my health^a^	64.1%	54.3%**
HPV would be a serious threat to my sex life^a^	69.6%	66.7%
HPV would make it difficult to find a long-term partner^a^	64.3%	65.7%
I would tell my sexual partner if I had HPV^a^	94.9%	87.6%*
If I had HPV I would be at risk for transmitting it to others	68.0%	64.8%
I would need the HPV vaccine if I had a high number of sexual partners^a^	70.1%	57.2%**
I would need the HPV vaccine if I had multiple sexual partners^a^	73.7%	62.9%**
I would need the HPV vaccine if I had a family history of cervical cancer^a^	60.3%	N/A
I would need the HPV vaccine if I regularly used a condom when engaging in sexual activity	57.1%	47.6%**
I would need the HPV vaccine if I engaged in sexual activity with a same sex partner	59.1%	46.7%**
I would need the HPV vaccine if I had a family history of liver cancer^a^	23.0%	21.9%
I would need the HPV vaccine if I had a steady long term partner^a^	57.5%	34.3%***
I would need the HPV vaccine if I smoked^a^	26.8%	31.4%
I would need the HPV vaccine if I engaged in sexual activity with a partner of the opposite sex	59.1%	70.5%**
I would need the HPV vaccine if I engage in unprotected sexual activity	53.6%	61.9%**
The HPV vaccine has significant side effects^b^	21.4%	17.1%
The HPV vaccine was thoroughly tested^b^	45.3%	51.4%*
The HPV vaccine is likely to cause health problems^b^	12.0%	11.4%
I could get HPV from the vaccine^a^	16.5%	20.9%*
I am concerned my family would find out if I got the HPV vaccine^a^	24.9%	26.7%
I am concerned my friends would find out if I got the HPV vaccine^a^	24.1%	25.7%
The HPV vaccine is an effective way to prevent HPV infection	59.0%	50.5%**
Overall, the HPV vaccine is safe^b^	56.8%	53.3%

^a^Survey item from Katz, Krieger, Roberto [20];

^b^Survey item from Marchand, Glenn, Bastani [21];

* = p < .05;

** = p < .01;

*** = p < .001

#### Vaccination prevalence and adoption stage

HPV vaccine uptake was reported by 47.3% of the females and 15.8% of the males (*p* < .001). Participants who were not vaccinated were asked to indicate their thoughts about getting vaccinated to determine which stage of the PAPM they were in at that point in time. The percent of participants in each stage indicated that most unvaccinated college students were in the earliest of the model’s stages as described in Table 3

**Table 3:** Precaution Adoption Process Model stage.

Please indicate which statement best indicates your thoughts about the HPV vaccination today?	Females	Males
Stages 1 and 2: *I never seriously thought about getting the HPV vaccination*.	62.9%	90.0%
Stage 3: *I have seriously thought about getting the HPV vaccination*, *but have not thought about it in past 6 months*.	11.2%	2.5%
Stage 4: *I have seriously thought about getting the HPV vaccination*, *but decided against it*.	18.1%	1.3%
Stage 5: *I am seriously thinking about getting the HPV vaccination sometime within the next 6 months*.	5.2%	5.0%
Stage 5, transitioning to Stage 6: *I plan to get the HPV vaccination within the next month*.	2.6%	1.3%

* = p < .05;

** = p < .01;

*** = p < .001

The vast majority of males (90%) indicated that they had never seriously thought about getting the HPV vaccination, but a significant portion of females reported this as well (62.9%). Overall, females were further along in the stages compared to males (*X*^*2*^ = 21.78(4); *p* < .001). Unvaccinated participants were further asked how likely they were to get the HPV vaccine within the next six months. Vaccination intention was low, with 51.7% of females and 52.5% of males reporting that they were “very unlikely” or “unlikely” to get the vaccine within the next six months.

### Factors associated with vaccination status

Logistic regression analysis was conducted to identify factors associated with vaccination status. A forward selection method based on changes in the likelihood ratio was utilized to determine the final set of predictors in the model. Factors associated with vaccination status included being offered the vaccine by a health care provider, believing that people who care about you think you should get the vaccine, and belief that the vaccine is likely to cause health problems. Approximately 87.1% of vaccination statuses (vaccinated yes/no) were predicted by this model described in Table 4.

**Table 4:** Factors associated with vaccination status.

					95% CI for Odds Ratio		
	*β* (SE)	Wald statistic	Df	*p*	Lower	Odds Ratio	Upper
Offered Vaccine	3.310 (0.454)	53.544	1	.000	27.64	11.36	67.23
Family/friends want you to get vaccine	1.018 (0.196)	27.011	1	.000	2.77	1.89	4.06
Belief that vaccine likely causes health problems	-0.546 (0.216)	6.423	1	.011	0.58	0.38	0.88
Constant	-4.683 (0.959)	23.825	1	.000			

CI = confidence interval; Hosmer & Lemeshow χ^2^(7) = 8.049, *p<0*.*328*; Nagelkerke *R*^*2*^ = 0.662; Model χ^2^(3) = 190.80, *p<0*.*001*

## Discussion

The Advisory Committee on Immunization Practices (ACIP) recommends catch-up HPV vaccinations for young adults who were not vaccinated during adolescence [5]. The results of this study are consistent with prior research which indicates that a significant portion of college-aged individuals, particularly males, are not yet vaccinated [3]. This study utilized the Precaution Adoption Process Model to understand college students’ perceptions toward vaccination completion. Students had moderate levels of knowledge of HPV causes and consequences, with health care providers and schools cited as the most common sources of information, but many lacked awareness of the vaccine or had never really considered it. These results indicate that most of the unvaccinated students are in Stage 1, unaware, and Stage 2, unengaged, of the Precaution Adoption Process Model (Table 1). Students reported awareness of HPV and awareness of common facts about HPV, however the mean knowledge score indicates that there are significant gaps in their knowledge about HPV, including risk and protective factors. This is important because previous research in female college students found that greater knowledge about HPV was associated with willingness to receive the vaccine [22]. Other studies have found a similar relationship between knowledge and HPV vaccination intention and uptake [23–24]. While 63.2% of the students indicated that they were in a relationship and 63.4% indicated that they have had sexual intercourse, students reported relatively low levels of perceived risk related to HPV. The results of the logistic regression analysis provide insight into factors associated with vaccination status that should be considered when developing HPV vaccination programs. Specifically, health care provider prompts and social support for vaccination were associated with vaccination, which are consistent with the interventions that move individuals from early stages of considering preventive behaviors to action according to the PAPM. This could be particularly valuable for males as relatively few had ever had a health care provider offer the vaccine to them.

To the best of our knowledge this is the first study to use the Precaution Adoption Process Model to evaluate where college students are in terms of decision making related to HPV vaccination. College campus health centers have a unique opportunity to significantly expand HPV vaccination coverage, thereby protecting the short and long-term health of this population. Understanding how to communicate to and engage students on this topic is essential to effectively develop interventions to motivate vaccination uptake.

Limitations of this study include the use of a cross-sectional survey design and self-report measures. Additionally, the sample includes only those who self-selected to participate. However, the final sample was representative of the university’s population. This survey instrument combined items from previous investigations of HPV in college students and added additional constructs. This exploratory study did not validate this instrument. Further research is needed to investigate the reliability and validity of the assessment. This study is unique in utilizing the PAPM to examine college students’ intentions, knowledge, and attitudes related to HPV and HPV vaccination.

## Conclusions

There are suboptimal levels of HPV vaccine coverage in college students in the United States [4]. Importantly, this study found that most male college students were not even aware that a vaccine was available and few had been offered the vaccine by health care providers. Colleges have an opportunity to fill this knowledge gap and to address the barriers identified in this study, including addressing concerns about the HPV vaccine, via health educational campaigns and activities. The strongest predictor of vaccination in this sample was the recommendation by a health care provider. Campus health centers have a unique opportunity to provide this recommendation by adopting routine protocols to offer HPV vaccination to all unvaccinated students. Students are usually required to have health insurance and are conveniently located to campus health centers, addressing cost and convenience barriers. Providing information about HPV susceptibility and about HPV vaccine availability, coupled with the easy access for vaccine administration, may provide an opportunity to increase HPV vaccine uptake in college students.

## Supporting information

S1 Fig Precaution Adoption Process Model. (TIF)

S1 File Survey instrument. (PDF)

S2 File Dataset. (XLSX)
