# Supplementary figures and images for: Human papillomavirus (HPV) vaccine knowledge, attitudes, and uptake in college students: Implications from the Precaution Adoption Process Model
Source: PLoS One. 2017 Aug 7;12(8):e0182266. doi: 10.1371/journal.pone.0182266 (PMC5546631; doi:10.1371/journal.pone.0182266)

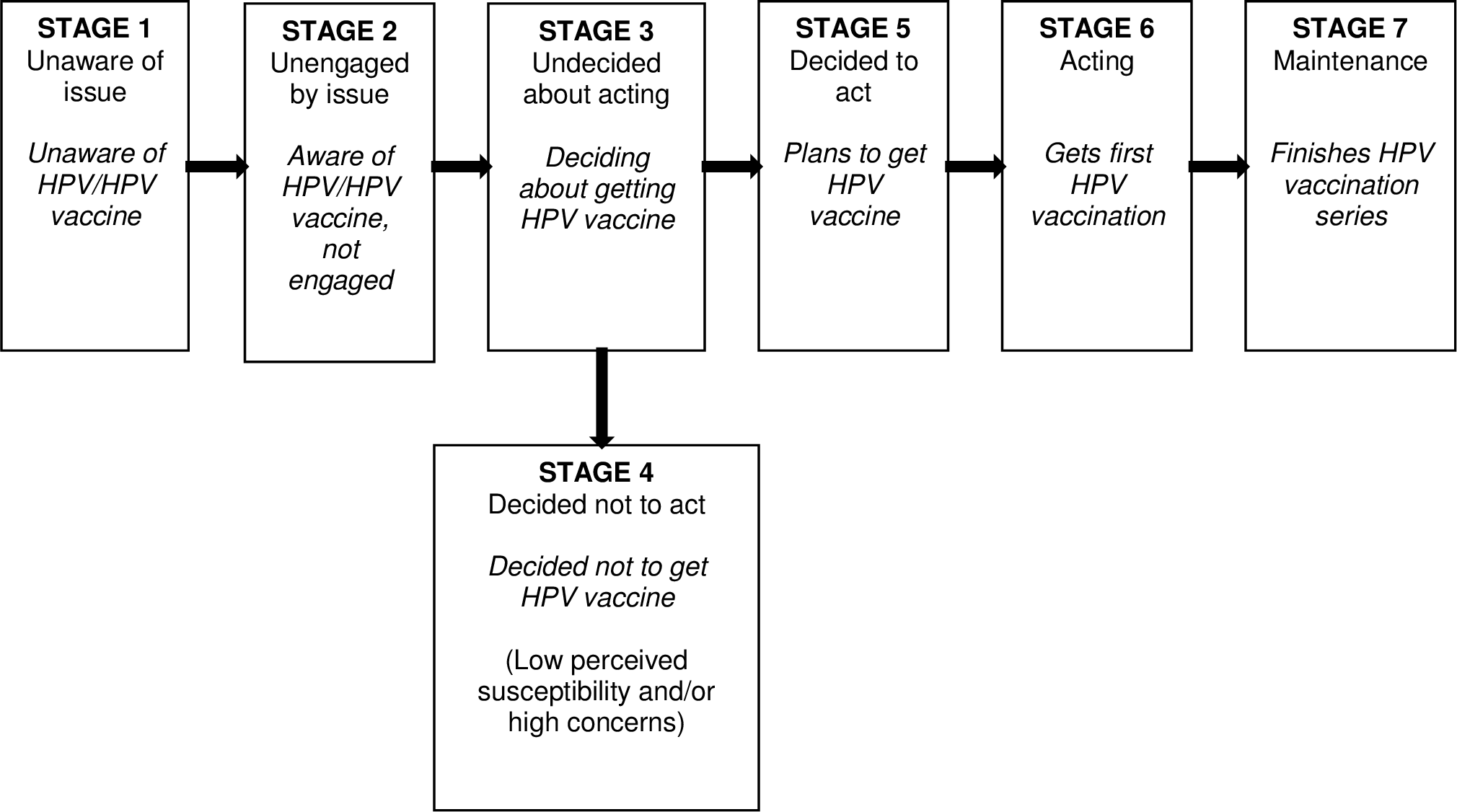

Supplement: S1 Fig — (TIF) [file pone.0182266.s001.tif]
