# Supplementary material for: Human papillomavirus (HPV) vaccine knowledge, attitudes, and uptake in college students: Implications from the Precaution Adoption Process Model
Source: PLoS One. 2017 Aug 7;12(8):e0182266. doi: 10.1371/journal.pone.0182266 (PMC5546631; doi:10.1371/journal.pone.0182266)
Supplement: S1 File — (PDF) [file pone.0182266.s002.pdf]

HPV Survey

The purpose of this survey is to learn about college students' knowledge and health beliefs concerning human papillomavirus (HPV). Your responses are completely anonymous; there is no way for anyone to connect your responses to you. You will have the option of entering a drawing for one of four $20 Amazon gift cards at the conclusion of the survey. Please answer the following questions to the best of your knowledge without looking up any answers. This survey will take about 10 minutes to complete. Please continue to start the brief survey.

What is your age?
- ❍ Under 18 years (1)
- ❍ 18 years old (11)
- ❍ 19 years old (2)
- ❍ 20 years old (3)
- ❍ 21 years old (4)
- ❍ 22 years old (5)
- ❍ 23 years old (6)
- ❍ 24 years old (7)
- ❍ 25 years old (8)
- ❍ 26 years old (9)
- ❍ 27 or more years old (10)

If Under 18 years Is Selected, Then Skip To Thank you for your interest in this s...

Display This Question:
    If What is your age? (U.S. Census 7 Categories) Under 18 years Is Selected

Thank you for your interest in this survey. You must be 18 years of age or older to participate.

If Thank you for your interest... Is Displayed, Then Skip To End of Survey

How do you identify your gender?
- ❍ Female (1)
- ❍ Male (2)
- ❍ Transgender (3)
- ❍ I prefer not to answer (4)

Are you Hispanic, Latino/a, or Spanish in origin?
- ❍ Yes (1)
- ❍ No (2)
- ❍ Uncertain (3)
- ❍ Prefer not to answer (4)

What is your racial or ethnic identification? (Select all that apply)
- ❑ Black or African American (3)
- ❑ White (6)
- ❑ Asian (2)
- ❑ American Indian or Alaska Native (1)
- ❑ Native Hawaiian or Other Pacific Islander (5)
- ❑ Other (7) ____________________
- ❑ Prefer not to answer (8)

What is your year in school?

- ❍ 1st year undergraduate (1)
- ❍ 2nd year undergraduate (2)
- ❍ 3rd year undergraduate (3)
- ❍ 4th year undergraduate (4)
- ❍ 5th year undergraduate (5)
- ❍ Not seeking a degree (6)
- ❍ Other (7)

What is your major? If you are a double or triple major, please list all of your majors.

Do you belong to or attend a church, synagogue, or any other religious organization?
- Yes (1)
- No (2)
- Prefer not to answer (3)

Which one of these statements is true for you?
- There have been times in my life when I did NOT have any health insurance (1)
- I have always had some kind of health insurance (2)
- I am uncertain about my history of health insurance (3)

Do you have a primary health care provider (i.e., a regular doctor)?
- Yes (1)
- No (2)
- Uncertain (3)

Do you rely on your parents when making a medical decision?
- Never (1)
- Rarely (2)
- Sometimes (3)
- Often (4)
- Always (5)

The following questions are about the human papillomavirus, also known as HPV. Please answer each item to the best of your knowledge.

	True (1)	False (2)
Genital warts are caused by HPV (1)	❍	❍
HPV can cause cervical cancer (2)	❍	❍
Abnormal pap tests may indicate that a woman has HPV (3)	❍	❍
HPV can cause penile cancer (4)	❍	❍
HPV is transmitted by skin-to-skin contact (5)	❍	❍
HPV infects both men and women equally (6)	❍	❍
HPV is sexually transmitted (7)	❍	❍
I can transmit HPV even if I don't have symptoms (8)	❍	❍
Most persons with HPV have no visible signs or symptoms (9)	❍	❍
HPV can lay dormant in the body for years without symptoms (10)	❍	❍
There is a vaccine available to prevent HPV infection (11)	❍	❍
Condoms are not effective in preventing HPV (12)	❍	❍
There is no cure for HPV (13)	❍	❍
Most adults are infected with HPV (14)	❍	❍
HPV infection among men is rare (15)	❍	❍
HPV can cause head and neck cancer (16)	❍	❍
HPV can cause anal cancer (17)	❍	❍
There is an HPV test for men (18)	❍	❍

I would need the HPV vaccine if:

	Strongly Disagree (1)	Disagree (2)	Neither Agree nor Disagree (3)	Agree (4)	Strongly Agree (5)
I had a high number of sexual partners (1)	❍	❍	❍	❍	❍
I had multiple sexual partners (2)	❍	❍	❍	❍	❍
I had a family history of cervical cancer (3)	❍	❍	❍	❍	❍
I regularly used a condom when engaging in sexual activity (10)	❍	❍	❍	❍	❍
I engaged in sexual activity with a same sex partner (4)	❍	❍	❍	❍	❍
I had a family history of liver cancer (5)	❍	❍	❍	❍	❍
I had a steady long-term partner (6)	❍	❍	❍	❍	❍
I smoked (7)	❍	❍	❍	❍	❍
I engaged in sexual activity with a partner of the opposite sex (8)	❍	❍	❍	❍	❍
I engaged in unprotected sexual activity (9)	❍	❍	❍	❍	❍

Please indicate your level of agreement with each statement.

	Strongly Disagree (1)	Disagree (2)	Neither Agree nor Disagree (3)	Agree (4)	Strongly Agree (5)
I would tell my sexual partner if I had HPV (1)	❍	❍	❍	❍	❍
HPV would be a serious threat to my sex life (2)	❍	❍	❍	❍	❍
HPV would make it difficult to find a long-term partner (3)	❍	❍	❍	❍	❍
HPV would be a severe threat to my health (4)	❍	❍	❍	❍	❍
I could get HPV from the vaccine (5)	❍	❍	❍	❍	❍
I am concerned my family would find out if I got the HPV vaccine (6)	❍	❍	❍	❍	❍
I am concerned my friends would find out if I got the HPV vaccine (7)	❍	❍	❍	❍	❍
I am at risk for getting HPV (8)	❍	❍	❍	❍	❍
I am likely to contract the HPV virus in my lifetime (9)	❍	❍	❍	❍	❍
If I had HPV I would be at risk for transmitting it to others (10)	❍	❍	❍	❍	❍

Before today, had you ever heard of the human papillomavirus (also known as HPV)?

- ❍ Yes (1)
- ❍ No (2)
- ❍ Uncertain (3)

If Yes Is Not Selected, Then Skip To Before today, had you heard of a vacc...

Display This Question:

If Before today, had you ever heard of the human papillomavirus (also known as HPV)? Yes Is Selected

How did you hear about HPV? (check all that apply)

- ❑ News programs (1)
- ❑ Family (2)
- ❑ Health Care Provider (3)
- ❑ School (11)
- ❑ Friends (4)
- ❑ TV (5)
- ❑ Radio (6)
- ❑ Newspaper (7)
- ❑ Internet (8)
- ❑ Magazine (9)
- ❑ Other (10) ____________________

Before today, had you heard of a vaccine for HPV?

- ❍ Yes (1)
- ❍ No (2)
- ❍ Uncertain (3)

Display This Question:

If Before today, had you heard of a vaccine for HPV? Yes Is Selected

How did you hear about the HPV vaccine? (check all that apply)

- ❑ News programs (1)
- ❑ Family (2)
- ❑ Health Care Provider (3)
- ❑ School (11)
- ❑ Friends (4)
- ❑ TV (5)
- ❑ Radio (6)
- ❑ Newspaper (7)
- ❑ Internet (8)
- ❑ Magazine (9)
- ❑ Other (10) ____________________

Have you ever been offered the HPV vaccine by your doctor or nurse?

- ❍ Yes (1)
- ❍ No (2)
- ❍ Uncertain (3)

Are you aware of any friends or family members who have gotten the HPV vaccine?

- ❍ Yes (1)
- ❍ No (2)
- ❍ Uncertain (3)

Please indicate your level of agreement with each of the following statements about the HPV vaccine.

	Strongly Disagree (1)	Disagree (2)	Neither Agree nor Disagree (3)	Agree (4)	Strongly Agree (5)
The HPV vaccine has significant side effects (1)	❍	❍	❍	❍	❍
The HPV vaccine was thoroughly tested (2)	❍	❍	❍	❍	❍
The HPV vaccine is likely to cause health problems (3)	❍	❍	❍	❍	❍
Overall, the HPV vaccine is safe (4)	❍	❍	❍	❍	❍
The HPV vaccine is an effective way to prevent HPV infection (5)	❍	❍	❍	❍	❍
People who care about me think I should get the HPV vaccination (6)	❍	❍	❍	❍	❍

Please indicate your current relationship status:
- ❍ Single and not dating (1)
- ❍ In a relationship with one person only (dating, engaged, married) (2)
- ❍ Dating more than one person (3)
- ❍ Prefer not to answer (4)

Have you ever had sexual intercourse?
- ❍ Yes (1)
- ❍ No (2)
- ❍ Uncertain (3)
- ❍ Prefer not to answer (4)

Have you ever been diagnosed with a STI or STD (sexually transmitted infection or disease)?
- Yes (1)
- No (2)
- Uncertain (3)
- Prefer not to answer (4)

Have you ever been diagnosed with HPV or genital warts?
- Yes (1)
- No (2)
- Uncertain (3)
- Prefer not to answer (4)

Display This Question:
    If Please indicate your sex: Female Is Selected

Have you ever been diagnosed with an abnormal Pap smear?
- Yes (1)
- No (2)
- Prefer not to answer (4)

Have you been vaccinated for HPV? There are currently two HPV vaccines. If you have ever received either the Gardasil or the Cervarix shots, then you have been vaccinated.
- Yes (1)
- No (2)
- Uncertain (3)

If No Is Selected, Then Skip To If answer NO to 28

How many of the HPV vaccine shots have you received?
- 1 (1)
- 2 (2)
- 3 (completed series) (3)
- Uncertain (4)

Display This Question:
If If yes, how many shots have you received? 3 (completed series) Is Not Selected
How likely are you to get the remaining shots to finish the vaccine series?
- Very Unlikely (1)
- Unlikely (2)
- Undecided (3)
- Likely (4)
- Very Likely (5)

Display This Question:
If Have you been vaccinated for HPV? There are currently two HPV vaccines. If you have ever received... No Is Selected
Please indicate which statement best indicates your thoughts about the HPV vaccination today?
- I never seriously thought about getting the HPV vaccination (1)
- I have seriously thought about getting the HPV vaccination, but I decided against it (2)
- I have seriously thought about getting the HPV vaccination, but have not thought about it in the past 6 months (3)
- I am seriously thinking about getting the HPV vaccination sometime within the next 6 months (4)
- I plan to get the HPV vaccination within the next month (5)

Display This Question:
If Have you been vaccinated for HPV? There are currently two HPV vaccines. If you have ever received... No Is Selected
How likely are you to get the HPV vaccine?
- Very Unlikely (1)
- Unlikely (2)
- Undecided (3)
- Likely (4)
- Very Likely (5)

Display This Question:

If How likely are you to get the HPV vaccine? Very Unlikely Is Selected

Or How likely are you to get the HPV vaccine? Unlikely Is Selected

If you are unlikely or very unlikely to get the HPV vaccine, please consider describing why are you unlikely to get the HPV vaccine?

THIS COMPLETES THIS SURVEY. You will be automatically redirected to a separate survey to indicate your interest in entering the RAFFLE to win one of four $20 gift certificates to Amazon.com. You will also be able to indicate if you are interested in participating in follow-up focus groups or interviews about this topic or to request a copy of the results once the study is completed. Remember, you are being redirected to a separate survey and in no way can your email be connected to your responses on this survey.
